# Taiwanese Vegetarians and Omnivores: Dietary Composition, Prevalence of Diabetes and IFG

**DOI:** 10.1371/journal.pone.0088547

**Published:** 2014-02-11

**Authors:** Tina H. T. Chiu, Hui-Ya Huang, Yen-Feng Chiu, Wen-Harn Pan, Hui-Yi Kao, Jason P. C. Chiu, Ming-Nan Lin, Chin-Lon Lin

**Affiliations:** 1 Medical Mission, Tzu Chi Foundation, Hualien, Taiwan; 2 Graduate Institute of Epidemiology and Preventive Medicine, National Taiwan University, Taipei, Taiwan; 3 Department of Family Medicine, Buddhist Dalin Tzu Chi Hospital, Dalin, Chiayi County, Taiwan; 4 Department of Biostatistics and Bioinformatics, Institute of Population Health Sciences, National Health Research Institutes, Miaoli County, Zhunan, Taiwan; 5 Institute of Biomedical Sciences, Academia Sinica, Taipei, Taiwan; 6 Department of Computer Science, University of British Columbia, Vancouver, Canada; 7 Department of Family Medicine, College of Medicine, Tzu Chi University, Hualien, Taiwan; 8 Department of Internal Medicine, Buddhist Hualien Tzu Chi Hospital, Hualien, Taiwan; 9 Department of Internal Medicine, College of Medicine, Tzu Chi University, Hualien, Taiwan; University of Barcelona, Faculty of Biology, Spain

## Abstract

**Introduction:**

Vegetarian diets have been shown to improve glucose metabolism and reduce risk for diabetes in Westerners but whether Chinese vegetarian diets have the same benefits is unknown.

**Methods:**

We evaluated the association between diet and diabetes/impaired fasting glucose (IFG) among 4384 Taiwanese Buddhist volunteers and identified diabetes/IFG cases from a comprehensive review of medical history and fasting plasma glucose.

**Results:**

Vegetarians had higher intakes of carbohydrates, fiber, calcium, magnesium, total and non-heme iron, folate, vitamin A, and lower intakes of saturated fat, cholesterol, and vitamin B12. Besides avoiding meat and fish, vegetarians had higher intakes of soy products, vegetables, whole grains, but similar intakes of dairy and fruits, compared with omnivores. The crude prevalence of diabetes in vegetarians versus omnivores is 0.6% versus 2.3% in pre-menopausal women, 2.8% versus 10% in menopausal women, and 4.3% versus 8.1% in men. Polytomous logistic regression adjusting for age, body mass index, family history of diabetes, education, leisure time physical activity, smoking and alcohol, showed that this vegetarian diet was negatively associated with diabetes and IFG in men (OR for diabetes: 0.49, 95% CI: 0.28–0.89; OR for IFG: 0.66, 95% CI: 0.46–0.95); in pre-menopausal women (OR for diabetes: 0.26, 95% CI: 0.06–1.21; OR for IFG: 0.60, 95% CI: 0.35–1.04); and in menopausal women (OR for diabetes: 0.25, 95% CI: 0.15–0.42; OR for IFG: 0.73, 95% CI: 0.56–0.95).

**Conclusion:**

We found a strong protective association between Taiwanese vegetarian diet and diabetes/IFG, after controlling for various potential confounders and risk factors.

## Background

The diabetes epidemic in Asia and particularly in China emerged simultaneously with increased meat consumption and higher proportion of energy intake from animal protein and fat [1]. Compared with Westerners, Asians tend to incur diabetes at a younger age and at a lower body mass index (BMI), possibly due to genetic susceptibility in combination with environmental exposures [2]. Vegetarian diets have been associated with a lower prevalence [3] and incidence [4] of diabetes among Seventh day Adventists. Previous clinical trials have shown vegetarian diets improve glycemic control [5] and insulin sensitivity [6]. Although several small studies reported lower glucose level and better insulin sensitivity in Taiwanese vegetarians than omnivores [7]–[9], no study thus far has examined whether a vegetarian diet protects against diabetes in Chinese ethnic Asian population, a high risk population that may incur diabetes despite having a normal BMI value [2]. Moreover, Asian diets tend to be lower in meat and higher in plant foods compared with Western diet. It remains unknown whether a diet completely avoiding meat and fish would further extend the protective effect of a plant-based diet. In addition, most studies on Asian vegetarians tend to compare vegetarians from religious groups with omnivores from the general population [7]. Religious and spiritual practices (a main determinant of vegetarian dietary practice in Asia) may be associated with social and emotional support which may confound health outcomes [10], [11].

This study examines within a Buddhist group, the cross-sectional association between vegetarian diet and two stages of impaired glucose metabolism – impaired fasting glucose (IFG) and diabetes.

## Methods

### The Tzu Chi Health Study

The Tzu Chi Health Study enrolled 6002 Taiwanese adults, of which 77% were Tzu Chi commissioners – a devoted group of volunteers of the Buddhist Tzu Chi Foundation who receive a free health examination every 2 to 3 years at one of four Tzu Chi hospitals. Tzu Chi Commissioners are required to abstain from alcohol, tobacco, and are encouraged to adopt a vegetarian diet for reasons of compassion and environmental conservation.

The current study was conducted at the Buddhist Dalin Tzu Chi Hospital between 2007 and 2009, where all participants received a health examination during an overnight stay at the hospital. Height was measured on a scale with participants standing erect. Body weight and body fat were measured on a Bioelectrical Impedance Analyzer (Tanita TBF-410). Waist circumference was measured at navel, with the participants standing. All measurements were performed with the participants wearing light clothes and without shoes. BMI was computed by dividing weight in kilograms by the square of height in meters. Venous blood was collected the next morning after an overnight fasting. Serum glucose was measured using the hexokinase glucose-6-phosphate dehydrogenase method (INTEGRA 800 system, Roche, USA).

Trained research dietitians interviewed each participant on demographic, lifestyle, diet, leisure time physical activity (LTPA), and medical history. Participants were identified as having a family history of diabetes if they reported one or more of their parents, grandparents, or siblings as having diabetes. Detailed diet was assessed through a 64-item food frequency questionnaire (FFQ), which had been validated in a subgroup of the present cohort [12], and the detail procedure of administering this FFQ had been reported previously [12]. Nutrients were calculated based on Taiwan's Food Composition Table [13]. Heme iron content was estimated using the following percentages of total iron: 65% for beef and lamb, 39% for pork, 26% for chicken and fish [14]. Only those who completely avoid meat, fish, and all animal flesh for at least one year up until entry into the study were considered vegetarians. Current smoker is defined as the use of any cigarette in the past 6 months. Alcohol drinking habit is defined as drinking of alcohol for at least once per week. The institutional review board at the Buddhist Dalin Tzu Chi Hospital approved the study, and all participants gave written informed consent.

### Disease ascertainment

Potential cases of diabetes were initially identified using self-reported history of diabetes ascertained from the baseline medical history questionnaire or having a fasting plasma glucose ≥7.0 mmol/L. Two physicians (HYH and MNL) subsequently confirmed the self-reported diabetes with the electronic medical records in Tzu Chi hospitals. For those who did not have medical records (28%), the physicians made telephone calls to confirm with the participants about their diabetes diagnosis. Participants who did not self-report a history of diabetes but had a fasting plasma glucose ≥7.0 mmol/L were regarded as having diabetes if one of the following criteria was further confirmed in medical record or with the participants in a telephone follow-up: (1) physician diagnosis of diabetes; (2) prescription of diabetes medication; (3) an additional fasting plasma glucose ≥7.0 mmol/L; (4) an additional HbA1C≥6.5%. Participants with one fasting plasma glucose ≥7.0 mmol/L but subsequent check-up showing no diabetes were classified as having IFG. Participants with fasting plasma glucose <5.6 mmol/L and 5.6–6.9 mmol/L were classified as normal and IFG respectively [15]. For participants who self reported diabetes during baseline questionnaire interview, the duration of their diabetes (how many years they have known to have diabetes up until entry to the study) was asked.

### Statistical analysis

After excluding 1377 non Tzu Chi commissioners, 35 participants who reported adopting vegetarian diet after diagnosis of diabetes, 10 participants with incomplete data on covariates, 267 participants with extreme average daily energy intakes (male: <3.3 MJ [800 kcal] or >16.7 MJ [4000 kcal], female: <2.1 MJ [500 kcal] or >14.6 MJ [3500 kcal]), and 13 participants whose diabetes status could not be confirmed (due to only one measurement of fasting blood glucose ≥7.0 mmol/L), a total of 4384 participants were included in the present analysis.

Demographic characteristics were compared using analysis of variance (continuous variables), or the Chi square test (categorical variables). Fisher's exact test was applied for categorical variables with a cell number less than 5. Dietary intake was compared using Wilcoxon two-sample test. Polytomous logistic regression was used to compare the outcomes of IFG and diabetes with normal glucose as the reference group, with adjustment for age, family history of diabetes, education, LTPA, BMI, smoking (men only), and alcohol (men only). All analyses were completed using SAS 9.2.

## Results

Table 1 shows the characteristics of participants with normal glucose, IFG, and diabetes. Diabetes individuals had the oldest age, highest BMI, waist circumference, body fat, and were the most likely to have family history of diabetes. Nearly all women were never smokers, while less than 5% of the men were current smokers. Among menopausal women, diabetes individuals reported more LTPA than those without diabetes.

**Table 1:** Characteristics of participants with normal glucose, impaired fasting glucose, and diabetes.

	Pre-menopausal women				Menopausal women				Men			
	Normal	IFG	Diabetes	P-value	Normal	IFG	Diabetes	P-value	Normal	IFG	Diabetes	P-value
n	866	75	16		1382	285	122		1253	266	119	
Age (years)	45±6	47±5	48±4	0.0001	58±7	60±7	62±8	<0.0001	54±10	58±9	59±8	<0.0001
BMI (kg/m^2^)	23±3	24±4	27±4	<0.0001	23±3	25±3	25±4	<0.0001	24±3	25±3	25±3	<0.0001
Waist (cm)	72±7	77±8	83±9	<0.0001	74±7	79±9	80±8	<0.0001	82±8	86±9	87±9	<0.0001
Body fat (%)	29±6	32±8	35±7	<0.0001	29±6	31±7	32±7	<0.0001	21±5	22±6	22±5	0.0004
Family history of diabetes	35%	33%	63%	0.068	28%	28%	63%	<0.0001	24%	27%	58%	<0.0001
Education												
Elementary or lower	9%	12%	44%	<0.0001*	39%	50%	58%	<0.0001	17%	23%	16%	0.0093
Secondary	65%	77%	50%		46%	40%	29%		48%	50%	59%	
College or higher	26%	11%	6%		15%	10%	13%		35%	27%	25%	
Smoking												
Current	0%	0%	0%	0.15*	0%	0%	0%	0.013*	4%	3%	5%	0.56
Past	2%	0%	0%		1%	1%	0%		33%	29%	36%	
Never	98%	100%	100%		99%	98%	100%		63%	67%	59%	
Alcohol												
Current	1%	4%	0%	0.004*	1%	0%	0%	0.0042*	8%	10%	8%	0.69
Past	1%	0%	6%		1%	1%	1%		23%	23%	24%	
Never	97%	96%	94%		98%	99%	99%		70%	67%	68%	
LTPA per week												
0–30 minutes	49%	55%	50%	0.0005*	31%	33%	22%	0.023	29%	29%	29%	0.33
31–180 minutes	33%	31%	31%		34%	32%	28%		33%	28%	28%	
>180 minutes	18%	15%	19%		35%	35%	50%		37%	42%	43%	
Diet												
Vegetarian	37%	27%	13%	0.0006*	48%	39%	18%	<0.0001	23%	16%	13%	0.0014
Omnivore	63%	73%	88%		52%	61%	82%		77%	84%	87%	

Data are presented as either mean ± standard deviation or percent. IFG  =  impaired fasting glucose BMI  =  body mass index. LTPA  =  leisure time physical activity.

*Fisher's exact test.

Table 2 shows the crude prevalence of diabetes, IFG, and other characteristics of vegetarians and omnivores. Vegetarians had lower prevalence of diabetes and IFG than omnivores. All groups had average BMI≤25 kg/m^2^, and had lower average waist circumference than recommended for Asians (men: <90 cm, and women <80 cm) [16]. Among men, vegetarians were less likely to ever use alcohol or tobacco.

**Table 2:** Crude prevalence of impaired glucose metabolism and other characteristics of vegetarians and omnivores.

	Pre-menopausal women			Menopausal women			Men		
	Vegetarians	Omnivores	P-value	Vegetarians	Omnivores	P-value	Vegetarians	Omnivores	P-value
n	343	614		792	997		349	1289	
Impaired glucose metabolism									
Diabetes	0.6%	2.3%	0.0006*	2.8%	10%	<0.0001	4.3%	8.1%	0.0014
Impaired fasting glucose	5.8%	9.0%		14%	18%		12%	17%	
Age (years)	46±5	45±6	0.0071	59±8	58±7	0.25	55±9	55±10	0.14
BMI (kg/m^2^)	23±3	23±3	0.023	23±3	24±3	<0.0001	23±3	24±3	<0.0001
Waist (cm)	72±7	73±8	0.0083	75±8	76±8	0.0008	81±8	84±8	<0.0001
Body fat (%)	28±5	30±7	<0.0001	28±6	31±6	<0.0001	19±5	22±5	<0.0001
Education									
Elementary or lower	10%	10%	0.90	44%	41%	0.35	19%	17%	0.65
Secondary	67%	65%		42%	45%		50%	49%	
College or higher	24%	25%		14%	14%		31%	34%	
Family history of diabetes	34%	36%	0.50	27%	33%	0.0097	28%	27%	0.85
Smoking									
Current	0%	0.5%	0.043*	0%	0%	0.09*	0%	5%	<0.0001*
Past	2%	1.5%		1%	1%		31%	33%	
Never	98%	98%		99%	99%		69%	62%	
Alcohol									
Current	1%	2%	0.012*	1%	1%	0.025*	1%	10%	<0.0001*
Past	1%	1%		1%	1%		26%	22%	
Never	98%	97%		98%	98%		72%	68%	
LTPA per week									
0–30 min	51%	49%	0.65	33%	28%	0.057	32%	29%	0.037
31–180 min	31%	33%		32%	35%		35%	31%	
>180 min	19%	17%		34%	37%		33%	40%	

Data are presented as either mean ± standard deviation or percent. BMI  =  body mass index. LTPA  =  leisure time physical activity.

*Fisher's exact test.

Overall, our study participants consumed a predominantly plant-based diet such that even the omnivores consumed little meat and fish (Table 3). Men consumed significantly more energy and most nutrients (except for calcium, folate, and vitamin C) than women. Vegetarians had a higher percent of energy intake as carbohydrate, and lower percent as fat and protein, while having higher intake of fiber, calcium, magnesium, non-heme iron, folate, vitamin A, and lower intake of saturated fat, cholesterol, and vitamin B12. Vegetarians also consumed more soy products, total and green leafy vegetables, nuts, whole grains; less tea; and a similar amount of dairy products and fruits, compared with omnivores. The majority (72% of men and 82% of women) did not report consumption of any sweetened beverage in the FFQ.

**Table 3:** Comparison of average daily dietary composition between vegetarians and omnivores as assessed by a food frequency questionnaire.

	Pre-menopausal women					Menopausal women					Men				
	Vegetarians		Omnivores		P-value	Vegetarians		Omnivores		P-value	Vegetarians		Omnivores		P-value
	Median	p25, p75	Median	p25, p75		Median	p25, p75	Median	p25, p75		Median	p25, p75	Median	p25, p75	
Energy (MJ)	7.17	5.41, 9.16	6.57	5.03, 8.50	0.0014	6.67	5.26, 8.20	6.15	4.73, 7.85	<0.0001	9.04	6.86, 11.47	8.89	6.92, 11.09	0.28
Protein (% energy)	12	11, 13	13	12, 15	<0.0001	12	11, 13	13	12, 15	<0.0001	11	10, 13	13	11, 14	<0.0001
Animal protein (g)	4	2, 7	13	8, 21	<0.0001	3	1, 7	12	7, 19	<0.0001	4	2, 8	19	11, 30	<0.0001
Plant protein (g)	46	33, 59	35	25, 46	<0.0001	42	33, 53	34	26, 44	<0.0001	55	41, 71	43	33, 55	<0.0001
Fat (% energy)	26	22, 32	29	24, 34	<0.0001	24	19, 29	26	21, 31	<0.0001	22	17, 27	25	19, 31	<0.0001
SFA (g)	9	6, 13	10	7, 15	0.0019	7	5, 11	8	6, 12	<0.0001	10	6, 14	12	8, 17	<0.0001
MUFA (g)	13	9, 19	14	10, 22	0.011	11	7, 17	12	8, 18	0.051	14	8, 20	17	11, 25	<0.0001
PUFA (g)	12	7, 19	11	7, 18	0.13	10	6, 15	9	5, 15	0.15	13	8, 21	13	8, 21	0.21
Carbohydrate (% energy)	63	57, 67	59	53, 65	<0.0001	65	60, 70	62	56, 67	<0.0001	67	62, 72	63	56, 69	<0.0001
Dietary fiber (g)	23	17, 30	19	14, 26	<0.0001	21	16, 29	19	14, 26	<0.0001	24	18, 33	20	15, 27	<0.0001
Cholesterol (g)	98	39, 155	146	87, 220	<0.0001	69	24, 111	104	58, 165	<0.0001	82	31, 151	159	100, 252	<0.0001
Potassium (g)	2.2	1.6, 3.0	2.1	1.5, 2.8	0.036	2.2	1.7, 2.9	2.1	1.6, 2.8	0.059	2.4	1.8, 3.1	2.3	1.7, 2.9	0.020
Calcium (mg)	602	408, 870	514	353, 751	0.0012	626	425, 931	572	380, 833	0.0005	645	445, 909	541	376, 781	<0.0001
Magnesium (mg)	284	210, 380	244	184, 321	<0.0001	290	211, 383	258	189, 352	<0.0001	325	231, 439	286	217, 380	<0.0001
Iron (mg)	13	9, 20	11	8, 16	<0.0001	12	9, 17	11	8, 15	<0.0001	14	10, 20	12	9, 16	<0.0001
Heme iron (mg)	0	0, 0	0.1	0.0, 0.3	<0.0001	0	0, 0	0.1	0.0, 0.2	<0.0001	0	0, 0	0.2	0.1, 0.4	<0.0001
Non-heme iron (mg)	13	9, 20	11	8, 16	<0.0001	12	9, 17	10	7, 15	<0.0001	14	10, 20	11	9, 16	<0.0001
Zinc (mg)	8.1	6.1, 12	7.7	5.7, 11	0.083	8.8	6.4, 14	8.3	6, 13	0.028	11.0	8.1, 14.3	10.4	7.9, 14	0.90
Thiamin (mg)	1.4	0.8, 2.8	1.0	0.6, 1.8	<0.0001	1.5	0.8, 3.0	1.2	0.7, 2.2	<0.0001	1.8	1.1, 3.3	1.3	0.8, 2.3	<0.0001
Riboflavin (mg)	1.0	0.7, 1.9	1.1	0.7, 1.7	0.88	1.1	0.7, 2.3	1.1	0.7, 2.2	0.75	1.1	0.8, 2.0	1.2	0.8, 1.9	0.43
Niacin (mg)	20	14, 31	20	13, 30	0.64	19	12, 31	20	13, 31	0.88	20	14, 31	22	15, 33	0.043
Vitamin B6 (mg)	1.2	0.9, 2.1	1.2	0.8, 1.8	0.52	1.2	0.9, 2.8	1.2	0.8, 2.4	0.43	1.4	1.0, 2.2	1.5	1.1, 2.3	0.17
Folate (µg)	451	308, 701	403	265, 598	0.0019	488	316, 713	433	288, 656	0.0003	493	321, 709	413	280, 607	<0.0001
Vitamin B12 (µg)	1.1	0.6, 3.5	2.7	1.5, 5.6	<0.0001	1.2	0.6, 7.1	2.9	1.4, 8.9	<0.0001	1.1	0.6, 3.2	4.1	2.2, 9.7	<0.0001
Vitamin C (mg)	169	119, 245	160	109, 224	0.078	165	116, 232	164	117, 238	0.85	176	123, 243	165	116, 222	0.017
Vitamin A (mg RE)	2.37	1.47, 3.48	2.04	1.21, 3.22	0.001	2.45	1.64, 3.73	2.18	1.39, 3.39	0.0001	2.70	1.61, 3.82	2.05	1.33, 3.17	<0.0001
Fish (g)	0	0, 0	5	1, 15	<0.0001	0	0, 0	7	2, 20	<0.0001	0	0, 0	15	4, 35	<0.0001
Fresh meat (g)	0	0, 0	11	2, 34	<0.0001	0	0, 0	7	1, 19	<0.0001	0	0, 0	20	7, 49	<0.0001
Processed meat (g)	0	0, 0	1	0, 5	<0.0001	0	0, 0	1	0, 3	<0.0001	0	0, 0	2	0, 6	<0.0001
Eggs (g)	16	6, 31	24	9, 32	<0.0001	7	2, 15	16	6, 24	<0.0001	15	4, 23	18	8, 31	<0.0001
Dairy products (g)	34	4, 115	41	2, 154	0.31	36	2, 144	50	1, 168	0.12	46	1, 161	46	1, 154	0.71
Soy products (g)	96	53, 176	68	30, 112	<0.0001	88	41, 144	52	23, 104	<0.0001	104	53, 176	63	27, 112	<0.0001
Total vegetables (g)	430	280, 650	380	250, 570	0.0013	440	290, 660	400	240, 590	<0.0001	470	300, 650	370	230, 550	<0.0001
Green leafy vegetables (g)	160	86, 200	110	71, 200	0.0014	200	100, 300	160	80, 200	<0.0001	170	80, 290	100	57, 200	<0.0001
Cruciferous vegetables (g)	86	43, 170	80	43, 150	0.34	86	43, 170	86	43, 160	0.28	100	43, 200	80	43, 150	0.0083
Other vegetables (g)	160	91, 270	150	78, 240	0.0019	140	75, 250	130	67, 220	0.0005	160	97, 260	130	70, 210	<0.0001
Fruits (g)	120	68, 240	120	60, 240	0.18	120	68, 240	120	64, 240	0.86	120	68, 240	120	60, 240	0.37
Nuts (g)	2	0, 9	1	0, 3	<0.0001	2	0, 7	1	0, 5	<0.0001	2	0, 8	2	0, 5	0.0006
Whole grain (% total grain)	28	12, 51	20	6, 42	<0.0001	32	12, 60	27	11, 52	0.02	20	8, 46	15	4, 37	<0.0001
Coffee (ml)	13	0, 129	21	0, 143	0.16	0	0, 25	0	0, 43	0.015	5	0, 36	8	0, 57	0.06
Tea (ml)	33	0, 286	100	8, 350	0.00013	0	0, 71	10	0, 214	<0.0001	86	0, 408	151	0, 504	0.0007
Sweeten beverage (ml)	0	0, 0	0	0, 8	0.02	0	0, 0	0	0, 0	0.28	0	0, 0	0	0, 12	0.0131

SFA  =  saturated fatty acid, MUFA  =  monounsaturated fatty acid, PUFA  =  polyunsaturated fatty acid, p25  =  25^th^ percentile, p75  =  75^th^ percentile.

Vegetarian diet is negatively associated with both IFG and diabetes in men, pre-menopausal, and menopausal women (Table 4). The protective association for diabetes is even stronger without controlling for BMI in men (OR: 0.43, 95% CI: 0.24–0.77), in pre-menopausal women (OR: 0.21, 95% CI: 0.05–0.97), and in menopausal women (OR: 0.23, 95% CI: 0.14–0.37). This association in pre-menopausal women was no longer statistical significant after adjusting for BMI as there were only 16 cases of diabetes in this group. BMI is highly correlated with waist circumference (the correlation coefficients r for male: 0.84, female: 0.74) and body fat (r for male: 0.76, female: 0.84). When either waist circumference or body fat was adjusted instead of BMI, similar results were found (data not shown). Family history of diabetes is significantly associated with diabetes but not with IFG. In men, current smoker (versus never smoker) is associated with diabetes, though not significant due to a low number of current smokers. In women, higher education (versus elementary school) is negatively associated with diabetes.

**Table 4:** Polytomous logistic regression analysis of the association between diet and impaired glucose metabolism.

	Men				Pre-menopausal women				Menopausal women			
	IFG		Diabetes		IFG		Diabetes		IFG		Diabetes	
	OR	95% CI	OR	95% CI	OR	95% CI	OR	95% CI	OR	95% CI	OR	95% CI
Age	1.05	1.03, 1.06	1.08	1.05, 1.10	1.10	1.04, 1.16	1.08	0.96, 1.22	1.03	1.01, 1.05	1.08	1.05, 1.11
BMI	1.12	1.07, 1.17	1.14	1.07, 1.22	1.13	1.05, 1.20	1.25	1.11, 1.42	1.14	1.10, 1.19	1.13	1.07, 1.20
Family history of diabetes vs none	1.29	0.94, 1.76	5.15	3.42, 7.75	0.92	0.55, 1.54	3.54	1.20, 10.44	1.01	0.75, 1.36	5.19	3.40, 7.93
Vegetarian vs omnivorous diet	0.66	0.46, 0.95	0.49	0.28, 0.89	0.60	0.35, 1.04	0.26	0.06, 1.21	0.73	0.56, 0.95	0.25	0.15, 0.42
Education												
Secondary vs elementary or lower	1.06	0.73, 1.53	1.65	0.93, 2.93	1.42	0.65, 3.11	0.26	0.08, 0.84	0.88	0.66, 1.19	0.56	0.35, 0.89
College or higher vs elementary or lower	0.87	0.58, 1.32	1.15	0.60, 2.19	0.60	0.21, 1.67	0.10	0.01, 0.89	0.68	0.43, 1.07	0.65	0.35, 1.22
LTPA per week												
31–180 min versus 30 min or less	0.82	0.57, 1.17	0.82	0.49, 1.39	0.79	0.46, 1.38	0.74	0.22, 2.49	0.91	0.66, 1.26	1.07	0.62, 1.86
> = 180 min vs 30 min or less	0.88	0.63, 1.24	0.87	0.53, 1.43	0.58	0.28, 1.18	0.93	0.22, 3.91	0.82	0.59, 1.13	1.52	0.91, 2.52
Smoking												
Current smokers vs never	0.67	0.31, 1.43	1.36	0.52, 3.52	-	-	-	-	-	-	-	-
Past smokers vs never	0.71	0.51, 0.99	1.01	0.64, 1.60	-	-	-	-	-	-	-	-
Alcohol drinking												
Current drinkers vs never	1.38	0.85, 2.26	0.88	0.41, 1.87	-	-	-	-	-	-	-	-
Past drinkers vs never	1.23	0.86, 1.76	1.00	0.60, 1.67	-	-	-	-	-	-	-	-

IFG  =  impaired fasting glucose. OR  =  odds ratio. BMI  =  body mass index. LTPA  =  leisure time physical activity.

As diabetes patients are often advised to change diet and lifestyle, we conducted a sensitivity analysis excluding those who self reported history of diabetes at baseline (n = 216), and counted only diabetes cases that were newly detected in this present study (n = 41). We found a similar protective association between vegetarian diet and diabetes in menopausal women (OR: 0.34, 95% CI: 0.12–0.95). The results were insignificant in men and pre-menopausal women due to limited number of cases of new diabetes.

## Discussion

In this Buddhist population consuming a plant-based diet with little meat and fish, true vegetarians who completely avoid animal flesh, while eating more soy, vegetables, nuts and whole grain, have lower odds for IFG and diabetes, after accounting for various confounders, risk factors, and BMI. The protective association is consistent in men and women although the association in pre-menopausal women was not significant due to a small number of diabetes cases.

Our result is consistent with the Adventist Health Study 2 (AHS-2), which found lower prevalence [3] and incidence [4] of diabetes in vegetarians. Similar to AHS-2 [17], we found vegetarians consumed a higher percentage of energy as carbohydrate, a lower percentage as fat and protein, and higher levels of plant protein, fiber, iron, and magnesium compared with omnivores in respective studies. We did not analyze subtypes of vegetarians (vegan, lacto-ovo-, or pesco-), as the AHS-2 did, since most of our vegetarians were of lacto-ovo type, with a small number of vegans (n = 69), and there were no cases of diabetes found within the vegan group. Consumption of eggs and dairy, however, was low, suggesting that the vegetarian diet in our population may resemble that of a vegan diet more than a typical Western lacto-ovo vegetarian diet when compared with AHS-2 [17] and EPIC-Oxford [18].

Despite similar or higher energy consumption, our vegetarians had lower BMI than omnivores. A similar finding is also observed in AHS-2 [3], [17]. Energy content of foods estimated by Atwater factors (for food composition tables and food labels) may not accurately reflect the actual energy utilized by the body due to the complexity in human digestion, and the variation in bioavailability as influenced by cooking and food processing methods, cell wall structures, and microbiome of the individual intestinal track [19]–[21].

The association between meat and diabetes has been reported recently in a large prospective study of European adults and a previous meta-analysis of prospective cohorts [22], [23], but not in a Shanghai study [24]. Although the Shanghainese population has a more similar ethnicity, the diabetes ascertainment depended on self-reported data; this could be a limitation for caution because the percent of undiagnosed diabetes (64% in urban China, 2000 to 2001) [25] is much higher than most Western countries (29% for the US, NHANES 1999–2000) [26]. Heme iron in meat had been suggested as a potential mediator leading to diabetes, as iron overload produces oxidative stress and induces insulin resistance [27], [28]. Although vegetarians had higher iron intake in both our study and in AHS-2, the iron is of non-heme form, which is absorbed to a lesser degree than that of heme iron from meat [29].

The higher intake of green leafy vegetables and magnesium may potentially contribute to the protective association between vegetarian diet and diabetes. Studies suggest that consuming more leafy green vegetables and a greater variety of fruits and vegetable are associated lower risk of diabetes in Europeans [30], [31]. A dietary pattern characterized by fruits, vegetables, and soy has also been associated with lower risk of diabetes in non-smoking Chinese [32]. A meta-analysis found magnesium to be protective of diabetes, and suggested that magnesium deficiency may induce insulin resistance [33].

### Strength and limitation

While our findings suggest a negative association between a vegetarian diet and diabetes/IFG, the temporal association is unclear due to the cross-sectional nature of the study. Although we have accounted for several confounders in our models, it is likely that other residual confounders still remain. The null association between LTPA and diabetes may have been influenced by reverse causation, as diabetes individuals may have increased physical activities in order to manage their disease. The measurements of body fat by Bioelectrical Impedance Analysis should be interpreted with caution as it has a poor accuracy for estimating absolute body composition [34].

The current study also has several strengths. The questionnaires were interviewed instead of self-administered – this enabled us to clarify questions, engage participants, and minimize potential inaccuracy due to fatigue or missing data. In addition, our study participants were relatively homogenous with similar religion and a very low proportion of smokers and alcohol-drinkers. This may minimize potential unadjusted confounders.

Our finding suggests that a vegetarian diet characterized by complete avoidance of meat and fish, and higher intake of soy products, vegetables, nuts, and whole grain may be more beneficial than an omnivorous diet with a moderate portion of meat and fish. Future follow-up on disease outcomes of this cohort will be needed to ascertain this finding and to delineate the impact of dietary components on the development of diabetes.
